# Modeling Tidal Marsh Distribution with Sea-Level Rise: Evaluating the Role of Vegetation, Sediment, and Upland Habitat in Marsh Resiliency

**DOI:** 10.1371/journal.pone.0088760

**Published:** 2014-02-13

**Authors:** Lisa M. Schile, John C. Callaway, James T. Morris, Diana Stralberg, V. Thomas Parker, Maggi Kelly

**Affiliations:** 1 Smithsonian Environmental Research Center, Edgewater, Maryland, United States of America; 2 Department of Environmental Science, Policy, and Management, University of California, Berkeley, California, United States of America; 3 Department of Environmental Science, University of San Francisco, San Francisco, California, United States of America; 4 Belle Baruch Institute for Marine and Coastal Sciences, University of South Carolina, Columbia, South Carolina, United States of America; 5 Department of Biological Sciences, University of Alberta, Edmonton, Canada; 6 Department of Biology, San Francisco State University, San Francisco, California, United States of America; MESC; University of South Alabama, United States of America

## Abstract

Tidal marshes maintain elevation relative to sea level through accumulation of mineral and organic matter, yet this dynamic accumulation feedback mechanism has not been modeled widely in the context of accelerated sea-level rise. Uncertainties exist about tidal marsh resiliency to accelerated sea-level rise, reduced sediment supply, reduced plant productivity under increased inundation, and limited upland habitat for marsh migration. We examined marsh resiliency under these uncertainties using the Marsh Equilibrium Model, a mechanistic, elevation-based soil cohort model, using a rich data set of plant productivity and physical properties from sites across the estuarine salinity gradient. Four tidal marshes were chosen along this gradient: two islands and two with adjacent uplands. Varying century sea-level rise (52, 100, 165, 180 cm) and suspended sediment concentrations (100%, 50%, and 25% of current concentrations), we simulated marsh accretion across vegetated elevations for 100 years, applying the results to high spatial resolution digital elevation models to quantify potential changes in marsh distributions. At low rates of sea-level rise and mid-high sediment concentrations, all marshes maintained vegetated elevations indicative of mid/high marsh habitat. With century sea-level rise at 100 and 165 cm, marshes shifted to low marsh elevations; mid/high marsh elevations were found only in former uplands. At the highest century sea-level rise and lowest sediment concentrations, the island marshes became dominated by mudflat elevations. Under the same sediment concentrations, low salinity brackish marshes containing highly productive vegetation had slower elevation loss compared to more saline sites with lower productivity. A similar trend was documented when comparing against a marsh accretion model that did not model vegetation feedbacks. Elevation predictions using the Marsh Equilibrium Model highlight the importance of including vegetation responses to sea-level rise. These results also emphasize the importance of adjacent uplands for long-term marsh survival and incorporating such areas in conservation planning efforts.

## Introduction

Sea levels are projected to rise by 20 to 180 cm over the next century [1]–[5]. With such a wide range of sea-level rise (SLR) predictions, tidal marsh resiliency is uncertain, as marshes may lose elevation at high rates yet remain stable at lower rates. We define resilience as the degree to which an ecosystem can maintain structure and function while withstanding chronic disturbance, in this case increased inundation [6]. Historically, tidal marshes have responded to increases in sea level by accreting sediment, which is affected by feedbacks between mineral [7]–[9] and organic matter input [10]–[13], and upland migration [14]–[16]. However, with projected increases in sea level, reductions in suspended sediment concentrations that drive mineral accretion [17]–[19] and decreased plant productivity with increased inundation [20], [21], uncertainties exist as to whether marshes will be able to maintain vegetated elevations. Furthermore, land-use change on adjacent upland habitat, including construction of levees, have restricted opportunities for migration [22]–[25], likely reducing marsh resiliency with projected SLR.

An array of marsh accretion models have been used to predict marsh responses to SLR, but there are trade-offs between obtaining local-scale predictions using detailed mechanistic models that include feedbacks between mineral and organic matter inputs and modeling landscape-level responses of marshes, including upland migration, at a coarser scale [26]. Many modeling efforts have sought to examine how tidal marsh elevations respond to changes in inundation, suspended sediment concentrations, and/or organic contribution due to predicted SLR (for detailed model reviews see [27], [28]), and more recent work has examined the impacts of increased temperature [29] and links to carbon sequestration potential [30]. Some modeling efforts have utilized a hybrid approach, merging results from mechanistic elevation-based models with digital elevation models to examine projections at site and landscape levels [31], [32]. However, hybrid approaches thus far have only mechanistically modeled the mineral contribution to marsh accretion and have not incorporated processes that affect the organic contribution to accretion, or interactions between mineral and organic matter contributions. Multiple studies have identified the importance of below-ground biomass contribution to vertical accretion [33], sustainability of marsh soils [34]–[36], and resiliency to increases in SLR [37], [38]. Therefore, it is valuable to integrate these feedbacks of vegetation with inundation, elevation, and sediment supply into a hybrid modeling approach [11], [12].

Across an estuarine landscape from salt to freshwater marshes, the contribution of mineral inputs and organic matter to accretion can vary depending on tidal marsh location within the estuary. In salt marsh communities where plant productivity is low, accretion is often dominated by mineral matter accumulation because of high levels of mineral sediment input and tidal energy [18], [39]. As freshwater influence increases and tidal energy decreases, plant productivity increases and accretion usually is dominated by peat accumulation [40]. As a result of these differing influences on marsh accretion, a model that incorporates the shifting importance of plant productivity and suspended sediment concentrations across a salinity gradient would more accurately represent marsh dynamics across the estuary.

In this study, we incorporated a rich dataset of above- and belowground plant productivity and physical characteristics across tidal marshes spanning a salinity gradient into a mechanistic elevation-based model, the Marsh Equilibrium Model version 3.76 (MEM; [11], [30], [41]). Model results were then applied to a high spatial resolution LiDAR-based digital elevation model to project changes in marsh elevation and extent, including upland migration, under a variety of SLR and suspended sediment concentration scenarios. The MEM incorporates a long history of field experiments and models that demonstrate how marsh elevation influences plant productivity, which in turn has a positive feedback on the rate of accretion [10]–[12], [30], [41]–[44]. Building upon a soil cohort model approach [41], [45], [46], the MEM lends itself to calibration against field-based vertical and mass-based accumulation rates using ^137^Cs and ^210^Pb dating techniques [39]. Combining a simple spreadsheet-based model interface with a fast processing time, the MEM is accessible for a broad array of end-users. Additionally, the MEM can be run using upland elevations that are not currently inundated to examine the timing and extent of marsh migration with a given rate of SLR.

The hybrid modeling approach used in this study builds upon the work of Stralberg et al. [32]. Using a one-dimension accretion model, Marsh98 [7], [47], [48], and regionally applied fixed organic accretion rates to digital elevation models of tidal marshes of San Francisco Bay Estuary, California, USA, Stralberg et al. [32] assessed tidal marsh sensitivity to changes in sea level and suspended sediment concentration. With MEM, we are able to incorporate a more integrated marsh accretion modeling approach to examine the sensitivity of different tidal marshes to changes in rates of SLR and suspended sediment availability while incorporating the dynamic inputs and feedbacks of plant productivity. The key objectives of this study were to: 1) calibrate the MEM for four tidal marshes along a salinity gradient in the San Francisco Bay Estuary that differ in plant productivity, sediment availability, and landscape setting (island versus unobstructed adjacent upland habitat), and examine 2) the influence of plant productivity on modeled marsh resiliency, 3) marsh resiliency relative to changes in SLR rates and reduction in suspended sediment concentrations, and 4) the importance of adjacent upland habitat on marsh resiliency with SLR over 100 years.

## Methods

### Study Area

**Figure 1:**
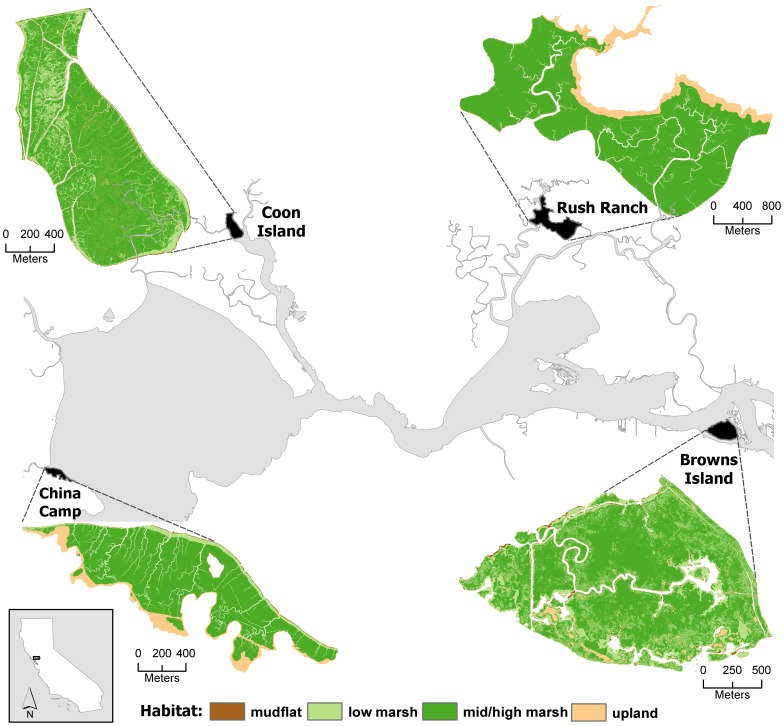
Field site locations and distribution of current habitat types.

We calibrated MEM at four historic tidal marshes in the San Francisco Bay Estuary (hereafter called Estuary), California, USA that span a salinity gradient from salt to nearly fresh water (Table 1, Fig. 1). All sites are 3–5 thousand years old and have been resilient over time with greatly varying sediment availability [40]. China Camp State Park (hereafter called China Camp) is a salt marsh. Coon Island is a high salinity brackish marsh. Rush Ranch Open Space Preserve (hereafter called Rush Ranch) is a low salinity brackish marsh. Browns Island is an oligohaline marsh at the confluence of the Sacramento and San Joaquin rivers. Detailed floristic data can be found in Vasey et al. [49]. Both Coon and Browns Island are islands with no upland transition and have a greater area of low marsh coverage compared to Rush Ranch and China Camp (Table 2; Fig. 1), although both have small upland areas within the site. Low marsh habitat at Rush Ranch and China Camp lines channel and bay edges and both sites have adjacent upland transitions zones (Table 2; Fig. 1). All sites are subject to semi-diurnal tides and are characterized by a Mediterranean-type climate with cool wet winters and dry warm summers. Direct human modifications to these sites are minimal (i.e., small levee construction, episodic dredge material deposits, mosquito ditches). All necessary permits were obtained for the study described below, which complied with all relevant regulations. Site access and data collection were permitted by the California Department of Fish and Game at Coon Island, Solano Land Trust and the National Estuarine Research Reserve at Rush Ranch, the East Bay Regional Parks District at Browns Island, and the California State Parks Service and the National Estuarine Research Reserve at China Camp.

**Table 1:** Site characteristics of marshes used for model calibration.

Site	Latitude	Longitude	water salinity (‰ NaCl)	plant productivity (g m^−2^)
China Camp	38°00′44″ N	122°29′35″ W	10–30	150–1750
Coon Island	38°11′44″ N	122°19′31″ W	3–24	245–1815
Rush Ranch	38°11′57″ N	122°01′53″ W	2–10	46–3300
Browns Island	38°2′21″ N	121°51′49″ W	0–5	160–3200

**Table 2:** Area (ha) of each habitat type in 2010 with percentage of coverage in parentheses.

	Site			
Habitat Type	ChinaCamp	CoonIsland	RushRanch	BrownsIsland
unvegetated	1.02 (1)	3.17 (2)	3.13 (1)	4.54 (2)
low marsh	9.11 (8)	30.15 (19)	13.27 (3)	74.21 (30)
mid/highmarsh	89.19 (78)	120.26 (76)	385.60 (84)	166.62 (67)
upland	14.69 (13)	4.70 (3)	54.43 (12)	4.61 (2)
total	114.02	158.28	456.43	249.98

### Marsh Equilibrium Model

The MEM incorporates both inorganic and organic inputs, described below, to model marsh accretion at a given elevation over a 100-year time period [30]. The MEM was calibrated initially at North Inlet located along the Atlantic Ocean that is dominated by *Spartina alterniflora*. This study is the first to calibrate MEM for Mediterranean-type marshes using sites along a salinity gradient. Physical inputs for the model include the initial rate of SLR, mean sea level, mean higher high water, suspended sediment concentration, and starting marsh elevation. The user also specifies a future sea level, which is reached after one century [50]. Biotic inputs include the minimum and maximum elevation for marsh vegetation, the peak aboveground biomass and the elevation at which it occurs, root to shoot ratio, organic matter decay rate, percent of refractory carbon, belowground turnover rate, and maximum rooting depth of 95% of the roots. The model assumes that plant productivity is constrained by upper and lower elevation limits and there is an optimum elevation for growth within the tidal frame [11], [20]. Two additional inputs, the trapping coefficient by which plants trap inorganic material and the sediment settling velocity, were assumed to hold constant across the marshes and were not changed from the initial model parameterization for North Inlet. The MEM incorporates a relationship between bulk density and percent organic matter; a curve calculated for San Francisco Bay tidal marshes [39] was used in lieu of the initial MEM relationship.

### Model Calibration

Five rates of SLR were chosen that spanned a spectrum of predicted rates. Sea level increased according to curves presented by the National Research Council [50]. We chose 24 cm/century, the rate of sea-level rise over the past century, to calibrate the model. Rates of 52 and 165 cm/century were consistent with those used in Stralberg et al. [32]. A rate of 100 cm/century is consistent with projections by the National Research Council [3], and 180 cm/century as the maximum published estimate of SLR at the time of this study [5].

Extensive field data were collected at each site in order to calibrate MEM (Table S1). For a minimum of two years, water depth was collected within a marsh channel at each site using a pressure transducer and mean tidal data were calculated relative to meters NAVD88. Since no published data on suspended sediment concentrations within the marshes were available, suspended sediment concentrations differed depending on location in the Estuary following Stralberg et al. [32]. At each site, we used three estimates representing what we considered to be high (current), middle (50% current), and low (25% current) concentrations. To be conservative in our estimates, we chose the current values to be the lowest reported in Stralberg et al. [32] (Table S1), since availability of suspended sediment within the Estuary has decreased since the large input of sediment from placer mining in Sierra Nevada mountain range in the 1800s [51] and is predicted to continue decreasing [17], [18]. Elevation surveys relative to NAVD88 were conducted using a real time kinematic GPS unit (horizontal and vertical accuracy of approximately two and three centimeters, respectively) to document the lowest and highest elevations used by marsh plants and to calibrate digital elevation models.

**Figure 2:**
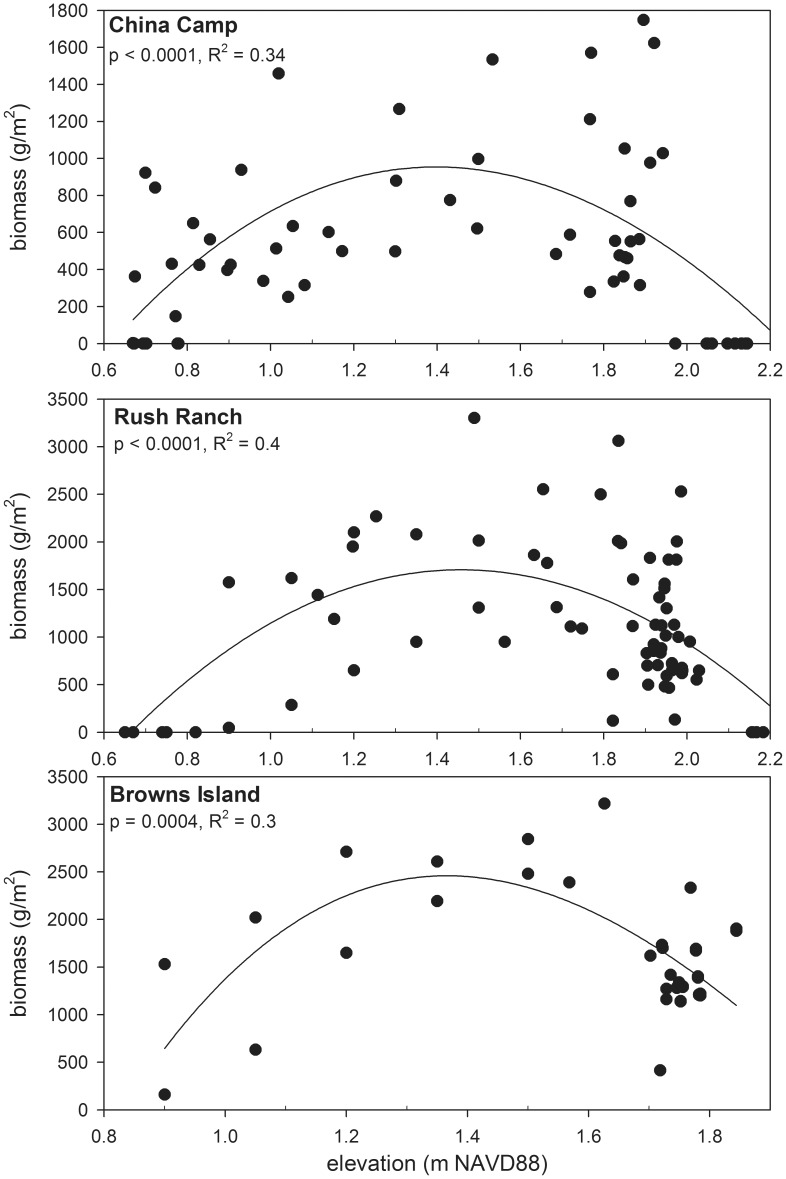
Community-level plant biomass with elevation. End of year above-ground biomass values across all vegetated elevations over multiple years at A) China Camp, B) Rush Ranch, and C) Browns Island.

Aboveground standing biomass representative of all vegetation types was collected at all sites on multiple occasions between 2004 and 2011 ([52], [53]; Schile, Parker, and Callaway unpublished data; Fig. 2; Fig. S1). Maximum biomass was measured at the end of the growing season as a surrogate for annual productivity, and surveys were targeted specifically to document productivity along elevation gradients. Data from a field experiment examining the productivity of two dominant plant species, *Schoenoplectus acutus* and *Schoenoplectus americanus*, at low marsh and mudflat elevations were also incorporated (Schile, Callaway, and Kelly, in review). We found a parabolic relationship between end of season plant biomass and elevation for Browns Island, Rush Ranch, and China Camp (Fig. 2), which fits the principle biotic assumption of MEM [11], [30], [54]. Based on the relationship between elevation and biomass and site knowledge, the elevation of peak biomass was determined. At Coon Island, where elevation data were not collected in tandem with biomass measurements, a histogram of biomass by plant species was created and the peak biomass elevation was chosen based on the species with the highest biomass and site knowledge of species occurrence (Fig. S1). Belowground biomass was collected between 2009 and 2011 at all sites (Schile, Callaway, and Kelly, in review; Parker, Callaway, and Schile unpublished data), and the depth of the rooting layer and root to shoot ratios were calculated. The organic decay rate and fraction of refractory carbon were not directly measured but were informed by percent organic carbon data at 40–50 cm soil depth from Callaway et al [39], the original MEM calibration for *S. alterniflora* marshes, and a litter decomposition study conducted over three years (Parker and Callaway unpublished data). The belowground turnover rate per year did not vary from the *S. alterniflora* MEM calibration.

We calibrated the model at each site using a SLR rate of 24 cm/century, which represents a hindcast of historic conditions over the last 100 years, and the current suspended sediment concentrations to test how accurately the MEM replicated marsh accretion rates as calculated by Callaway et al. [39]. We compared the model-generated average vertical accretion rates over 100 years to the accretion rates calculated using ^210^Pb dating of six soil cores collected across elevations at each marsh (Table S2). For calibration, MEM was run at the elevations where soil cores were taken. Model-generated sediment depth profiles of bulk density and percent organic matter were also compared to depth profiles generated from the cores (Fig. S2).

### Model Runs

We ran the model using all five rates of SLR and three suspended sediment concentration estimates, for a total of 15 scenarios per site. The MEM was run at elevations between 0 and 380 cm NAVD88 in 10 cm increments. The top elevation of 380 cm was chosen since it was the maximum upland elevation that would be inundated by a 180 cm increase in sea level. Results were interpolated evenly for every centimeter of elevation in between each model run.

### Spatial Analyses

A digital elevation model for each site was created using the 2009–2011 California Coastal Conservancy’s Coastal LiDAR Project data [55], which is more recent and at a higher spatial resolution than what was used in Stralberg et al. [32]. The elevation model was generated at a 1 m^2^ spatial resolution with the vertical datum NAVD88 GEOID09 model for orthometric heights. To account for the effects of dense vegetation on LiDAR-derived elevation models [56], elevations were adjusted between 0 and 70 cm lower when necessary based on comparison with comprehensive RTK elevation surveys across all sites, vegetation maps [57], and knowledge of vegetation distribution, height of live vegetation, and height of dense standing dead vegetation. Elevations were limited to 390 cm NAVD88 and lower to only include elevations relevant for the analysis, since both China Camp and Rush Ranch have unobstructed adjacent upland habitat that extends well above marsh elevations (Table 2). All values were rounded up to the nearest whole cm. The MEM works most accurately when applied to areas with laminar, not turbulent, flow [30]; therefore, a mask was digitized manually in ArcMap 10 [58] to remove tidal channels from the analysis.

At each site beginning with the initial elevation at time zero (2010), modeled elevations were compiled for runs that were 20, 50, 70 and 100 years into the future, corresponding to years 2030, 2060, 2080, and 2110, respectively. Using ArcMap Model Builder [58], modeled elevations from each time period were applied to the digital elevation model and then transformed relative to the local tidal datum using the equation: (marsh elevation – mean sea level)/(mean higher high water – mean sea level). We assumed that there was no change in the relationship between mean sea level and mean higher high water over time. In order to classify the elevations into marsh habitat type, we determined elevations for transitions between mudflat, low marsh, mid/high marsh, and upland habitat based on elevation surveys of current distributions of each habitat type relative to mean sea level (Table 3; see Fig. 1 for starting conditions). We chose not to differentiate between mid and high marsh habitat since distributions in Mediterranean-type climates do not always correspond with elevation alone [59], [60]. The area of each habitat type was calculated for every model scenario and time period.

**Table 3:** Elevation ranges normalized relative to local tides (m NAVD88) of habitat types at each site.

	Site			
	ChinaCamp	CoonIsland	RushRanch	BrownsIsland
unvegetated	< −0.3	< −0.3	< −0.3	< −0.3
low marsh	−0.3–0.7	−0.3–0.65	−0.3–0.74	−0.3–0.75
mid/high marsh	0.7–1.049	0.65–1.01	0.74–1.03	0.75–1.06
Upland	>1.049	>1.01	>1.03	>1.06

At the site level, we evaluated the stability of the distributions of current marsh habitats over time using the 24 cm/century SLR and current suspended sediment concentrations as a way of assessing model calibration/accuracy at that spatial scale. We assumed that marsh conditions have been relatively stable over the last 100 years and, as such, that the model results show little change in habitat distribution with the 24 cm/century rise.

### Plant Productivity and Elevation Feedbacks

More detailed comparisons with Stralberg et al. [32] to examine the influence of plant productivity on marsh resiliency were not possible because the two models used different digital elevation models. We could, however, examine the influence of plant productivity on wetland elevation by comparing modeled MEM results when suspended sediment concentrations were the same across all sites. Therefore, a suspended sediment concentration of 25 mg/L was used at all sites and elevations were compared using a century SLR of 180 cm.

## Results

### Model Calibration

Model-generated output for the 24 cm/century SLR scenario simulating historic conditions consistently matched core-based accretion rates and soil depth profiles of bulk density and percent organic matter (Table S2 & Fig. S2). Additionally, marsh habitat distributions changed little over 100 years with both the mid and high suspended sediment concentrations (Fig. S3), supporting historic observations of relatively stable tidal marshes within the Estuary over the last century.

### Mid and High Suspended Sediment Concentrations

**Figure 3:**
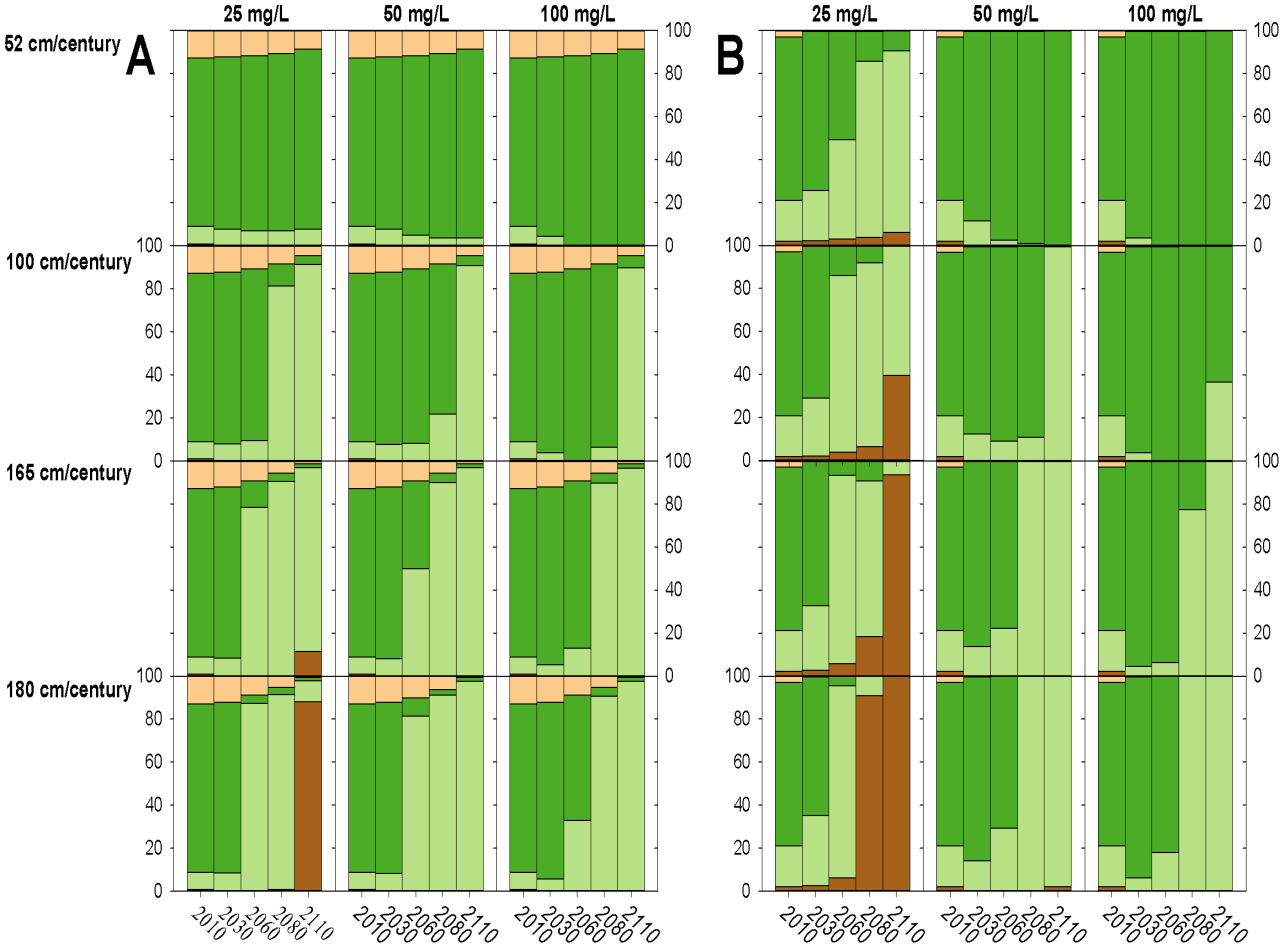
Change in habitat cover under all model scenarios at high salinity marshes. Modeled changes in habitat type cover over time for each suspended sediment concentration and sea-level rise scenario for A) China Camp and B) Coon Island, where pixels are color-coded by elevations indicative of unvegetated (brown), low marsh (light green), mid/high marsh (medium green), and upland (beige) elevations.

**Figure 4:**
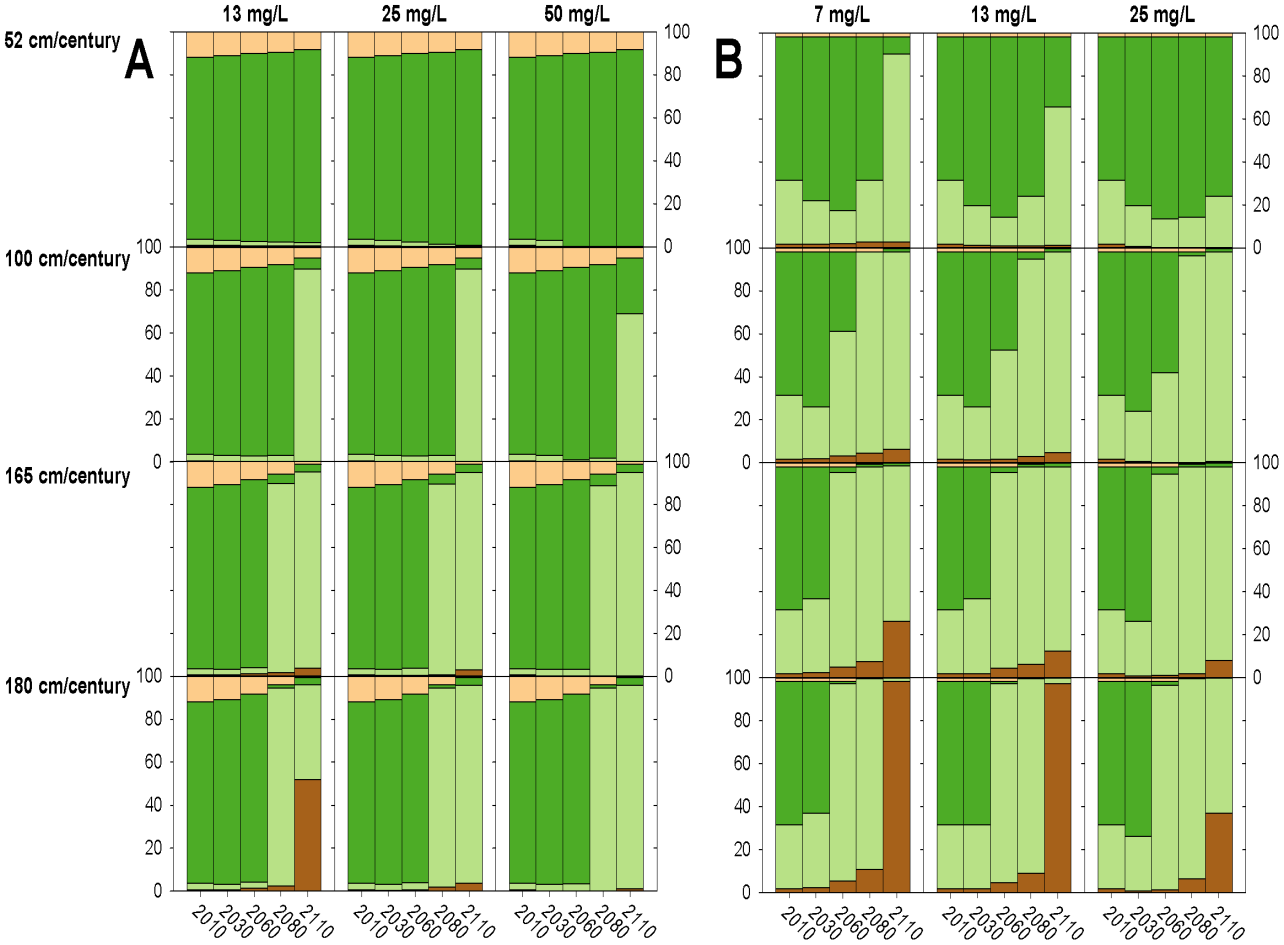
Change in habitat cover under all model scenarios at low salinity marshes. Modeled changes in habitat type cover over time for each suspended sediment concentration and sea-level rise scenario for A) Rush Ranch and B) Browns Island, where pixels are color-coded by elevations indicative of unvegetated (brown), low marsh (light green), mid/high marsh (medium green), and upland (beige) elevations.

**Figure 5:**
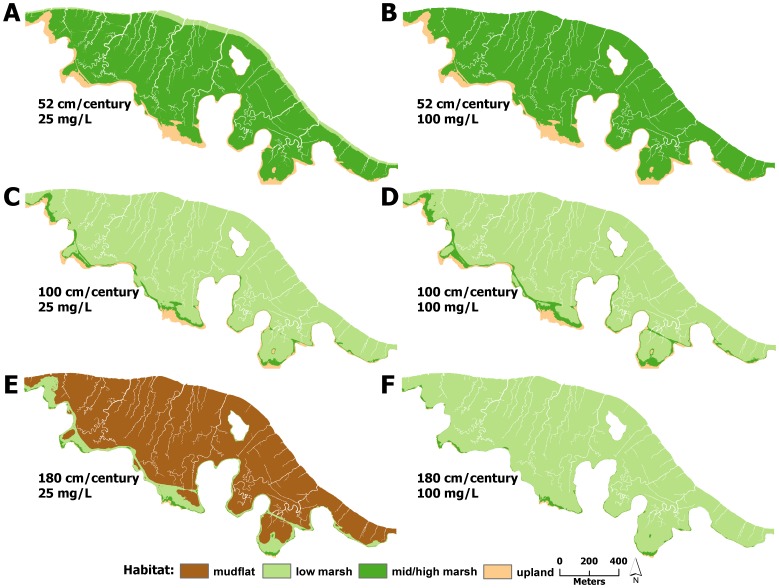
Habitat distributions at China Camp under different model scenarios. Distribution of modeled marsh habitat types in 2110 at China Camp with 52/century, 100 cm/century, and 180 cm/century sea-level rise at A,C,E) low and B,D,F) high suspended sediment concentrations, respectively.

**Figure 6:**
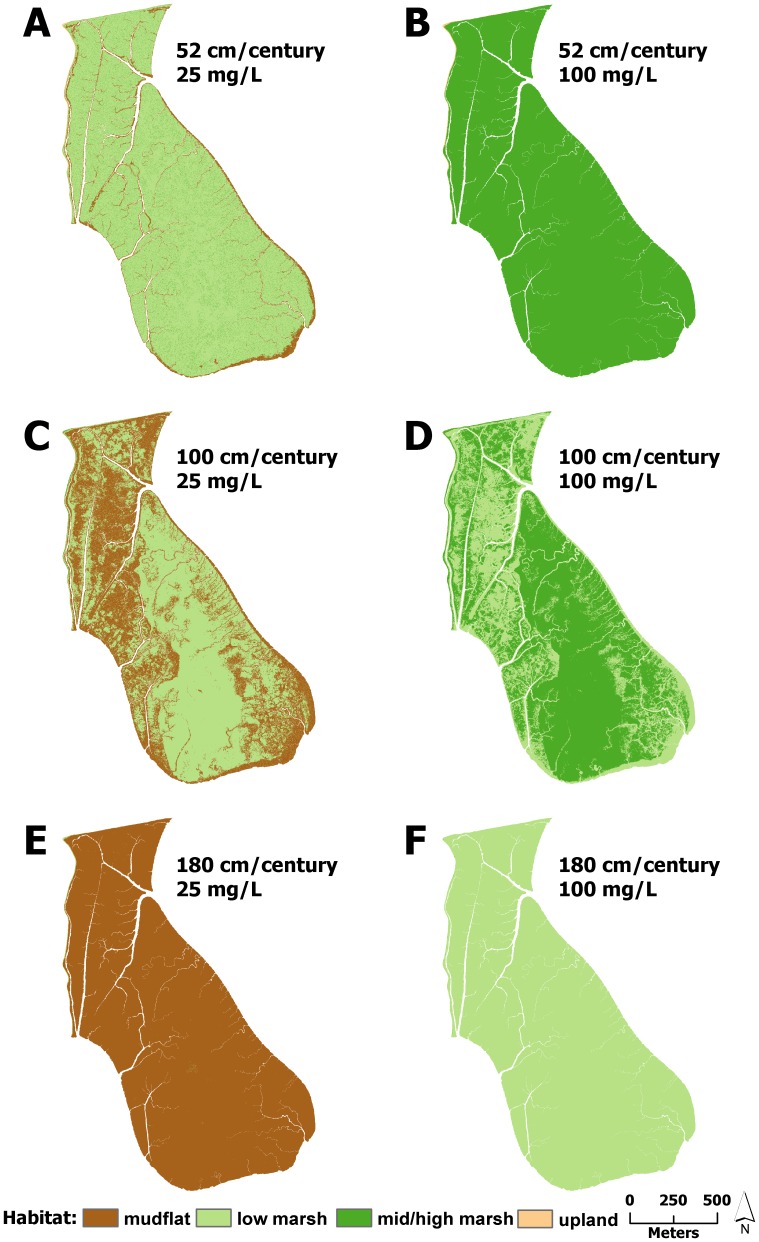
Habitat distributions at Coon Island under different model scenarios. Distribution of modeled marsh habitat types in 2110 at Coon Island with 52/century, 100 cm/century, and 180 cm/century sea-level rise at A,C,E) low and B,D,F) high suspended sediment concentrations, respectively.

**Figure 7:**
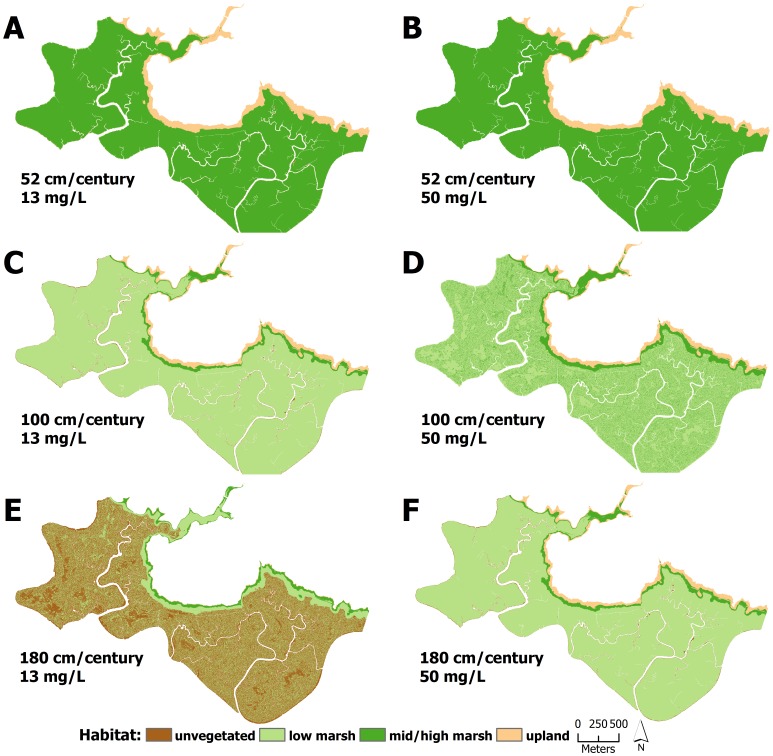
Habitat distributions at Rush Ranch under different model scenarios. Distribution of modeled marsh habitat types in 2110 at Rush Ranch with 52/century, 100 cm/century, and 180 cm/century sea-level rise at A,C,E) low and B,D,F) high suspended sediment concentrations, respectively.

**Figure 8:**
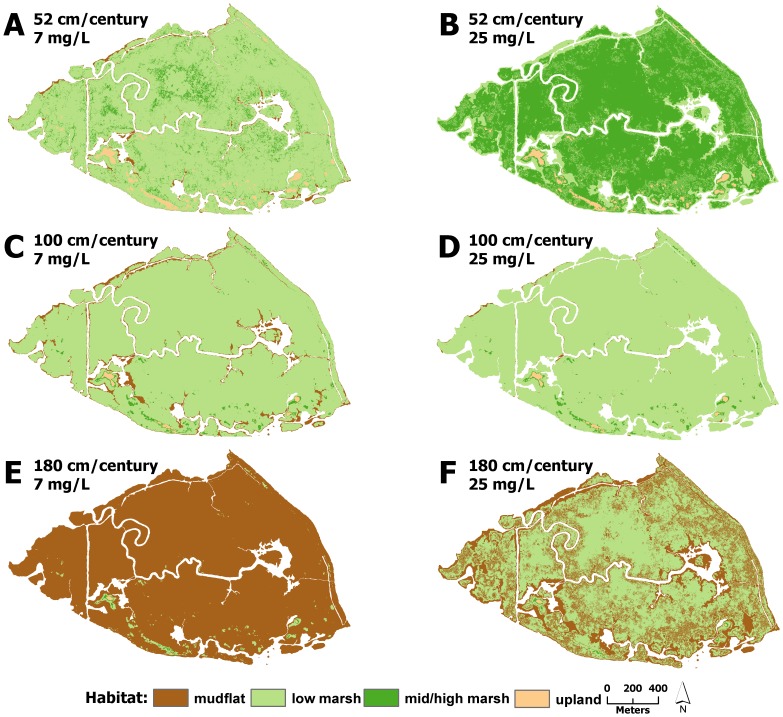
Habitat distributions at Browns Island under different model scenarios. Distribution of modeled marsh habitat types in 2110 at Browns Island with 52/century, 100 cm/century, and 180 cm/century sea-level rise at A,C,E) low and B,D,F) high suspended sediment concentrations, respectively.

Under mid and high suspended sediment concentrations, changes to modeled marsh habitat was strongly dependent on SLR (Figs. 3 & 4). Under the 52 cm/century SLR scenario, low marsh elevations tended to accrete to mid/high marsh elevations, covering between 74 and 99% of the marsh after 100 years (Figs. 3 & 4, Figs. 5a–8a). Conversely, elevations at all sites were indicative of low marsh habitat after 100 years under the 100 cm/century SLR scenario (Figs. 3 & 4, Figs. 5c–8c). All marshes responded similarly under the two higher (165 and 180 cm/century) SLR scenarios (Figs. 3 & 4), hence we only included maps for the 180 cm/century SLR rate (Figs. 5e–8e). With these high SLR rates, all marshes showed signs of elevation loss relative to sea level (Figs. 3 & 4, 5e–8e), but this occurred more rapidly at the island sites (Figs. 3b, 4b, 6e & 8e) that had lower initial starting elevations (50 years vs. 70 years). After 100 years, one island site (Browns Island) showed marked signs of drowning, as evidenced by the predominance of unvegetated habitat (97%; Fig. 4b, Fig. 8e). Less than 1% of mid/high marsh elevations remained at the two island sites after 100 years (Figs. 3b, 4b, 6e & 8e) and the only remaining mid/high marsh habitat at the other sites was in formerly upland areas (Figs. 5e & 7e).

### Low Suspended Sediment Concentrations

Under the lowest suspended sediment concentrations, differences among sites were more exaggerated. The upland-adjacent sites (China Camp and Rush Ranch) exhibited little response to SLR under the 52 cm/century SLR scenario (Figs. 3a, 4a, 5a & 7a), while island sites (Coon Island and Browns Island) shifted to low marsh-dominated systems after 70 years (Figs. 3b & 6a) and 100 years (Figs. 4b & 8a), respectively. With 100 and 165 cm/century SLR, low marsh elevations eventually dominated at the upland-adjacent sites, and marsh drowning began to occur after 100 years at the island sites (Figs. 3 & 4). With the highest SLR rate (180 cm/century), all sites were dominated by mudflat elevations and the only remaining vegetation occurred in former upland areas.

### Plant Productivity and Elevation Feedbacks

Under a 180 cm/century SLR rate and a suspended sediment concentration of 25 mg/l, a concentration that was modeled across all sites, the low salinity brackish wetlands with higher plant productivity largely maintained vegetated elevations on the marsh plain after 100 years (Fig. 8f; Fig. S4) compared to the lower productivity salt marshes (Figs. 5e & 6e), which had drowned after 100 years. Although the low salinity brackish sites were experiencing marsh drowning, the presence of highly productive vegetation reduced the rate at which elevations was lost.

## Discussion

### Effects of Plant Productivity on Marsh Resiliency

In this study, marsh resiliency to increased century SLR was greater when both the organic and mineral contributions to accretion were modeled mechanistically compared to Stralberg et al. [32], where only the mineral contributions were modeled (see http://data.prbo.org/apps/sfbslr/for maps of results). Incorporating vegetation response to inundation into marsh accretion models resulted in model predictions of more resilient marshes. Additionally, in MEM model runs where the suspended sediment concentration was the same across all sites, marsh resiliency was greater at sites with higher plant productivity. This finding coincides with evidence of the importance of organic matter contribution to accretion/elevation dynamics seen in previous field studies and experiments [36] and supports the inclusion of vegetation responses in future models of marsh accretion. Even though our sites had more diversity in dominant species and morphology than in North Inlet, our field data supported the MEM’s critical assumption of a parabolic relationship with productivity along an elevation gradient ([11], Fig. 2), and thus support the application of MEM across a wide variety of wetland ecosystems. Often, the collection of plant productivity data is labor intensive, particularly for below-ground biomass, yet these data are crucial in order to more accurately model marsh accretion [12], [38], particularly in lower salinity and freshwater sites with low rates of mineral matter input [29]. As demonstrated in this study, intensive field data collection and model calibration at select sites representative of wetlands across the estuarine salinity gradient will improve the app1ication of marsh accretion model at a broader estuary level, similar to Stralberg et al. [32]. Incorporation of spatial variation in wave exposure, local sediment delivery, human disturbance, and other environmental factors will improve further broad-scale spatial application of these types of models.

### Effect of Suspended Sediment Concentration on Marsh Resiliency

Modeled accretion rates did not keep pace with high rates of SLR when suspended sediment concentrations were low, a finding supported by Stralberg et al. [32] and other models [43]. Some of the highest suspended sediment concentrations occur in high salinity sites in San Francisco Bay, which may compensate for the decreased contribution of organic matter. Thus, a reduction in suspended sediment concentrations at the saltier sites resulted in an inability of the marsh to maintain current elevations with SLR; this effect was not as marked in the less saline sites. These results are corroborated by field studies that documented higher bulk density values in salt marshes; they require more mineral input to maintain elevations relative to SLR [61], [62]. A reduction in suspended sediment in the salt marshes resulted in an earlier conversion to low marsh elevations under the 100 cm/century SLR scenario whereas reduction in suspended sediment did not result in a large difference in modeled results for the lower salinity brackish sites, which had greater above- and below-ground primary production.

Although this study highlights the important role of organic matter contribution to marsh resiliency, the influence of suspended sediment was still apparent [63], particularly in the comparison of results from the two low salinity sites. Both Rush Ranch and Browns Island have comparable peak biomass (2,400 to 2,500 g/m^2^yr, respectively); however, more suspended sediment is available at Rush Ranch due to its location in the Estuary and water circulation patterns (Table S1). After 100 years at the highest rate of century SLR, Rush Ranch still maintained low marsh elevations across areas on the original marsh plain. Both the organic matter and mineral contributions are important to accretion at this site, and Rush Ranch appears to be the most resilient to SLR compared to the other sites under these modeled conditions. Browns Island had very little vegetated elevation after 100 years, all of which was in formerly upland habitat.

Suspended sediment inputs are clearly important to marsh resiliency, yet this parameter is the most uncertain of all model inputs. The majority of suspended sediment concentration measurements used to model tidal marsh resiliency has been made within open water bodies or large tributaries and data on sediment dynamics within marsh channels or on the marsh plain is largely unknown. Future model predictions will be improved with the incorporation of suspended sediment data that are collected across a marsh plain.

Intra and inter-annual variability in suspended sediment concentrations is a common occurrence in tidal marshes and other coastal ecosystems. The influence of storm-based sediment pulses on marsh accretion is well documented [64] but not taken into consideration with MEM, nor are changes in sediment concentrations over time. Furthermore, sediment concentrations in the Estuary and other estuaries worldwide have been dropping [51], [65]–[67] and are predicted to continue to drop [17], [18]. To account for these factors, we chose a variety of concentrations that might span current and future values, although our values may miss the extremes or overestimate concentrations at later time periods since suspended sediment concentrations are a fixed model input.

### Effect of Landscape Position and Elevation on Marsh Resiliency

A striking difference across sites was the availability of adjacent upland habitat for marsh migration. Under the highest SLR scenarios, mid/high marsh elevations were entirely restricted to what was initially upland habitat (Figs. 5e, 6e, & 8e). The island sites that lacked extensive upland habitat either had no mid/high marsh habitat after 100 years or were mostly unvegetated. As such, island sites appear to be less resilient under accelerated SLR, regardless of plant productivity and suspended sediment concentration. Management and conservation efforts for island marshes might require more intensive actions, such as dredge spoil application or sediment ‘seeding’, to help support marsh resiliency to increased rates of SLR. In tidal marshes that do have adjacent upland habitat, key efforts should be implemented to secure and protect these habitats to allow for marsh migration.

Initial marsh plain elevation played a role in marsh resiliency at the onset of increased SLR rates but the net result after 100 years was similar across sites. Both island sites had starting elevations that, on average, were lower than the other sites and had a broader coverage of low marsh habitat (Table 2; Fig. 1). Across all SLR scenarios, this initially translated into an increase in accretion rates in low marsh habitats resulting in elevations characteristic of mid/high marsh habitat, which has been documented in other modeling studies [11], [29], [44] and field studies [33], [68]. However, the marshes were unable to keep pace with continued increase in sea level, and the elevation shifted towards low marsh elevations. These shifting habitat patterns over time were not apparent at the upland-adjacent sites, where low marsh elevations are currently restricted to thin bands along channel edges. Both of these upland-bordering sites have broad marsh plains with relatively uniform elevations and large-scale shifts in habitat type occurred rather abruptly when the threshold points were crossed. Because marshes may differentially respond to accelerated SLR due to different initial elevation distributions, understanding these responses is critical for researchers and site managers in assessing the relative magnitude and timing of marsh changes.

### Marsh Equilibrium Model

There are multiple advantages to using MEM for modeling marsh accretion over time. First, the spreadsheet-based format enabled easy accessibility, transferability, and a fast processing time; a web-based version of a different model version is also available: http://jellyfish.geol.sc.edu/model/marsh/mem.asp. Second, MEM mechanistically models both the individual contributions of and feedbacks between mineral and organic matter input to accretion. This work builds upon Stralberg et al. [43], which only mechanistically modeled mineral accretion. Third, we were able to compare results to historic accretion data from dated soil cores with model outputs, examining mass-based mineral accretion, accretion rates, and soil profiles of bulk density and percent organic matter ([39]; Table S2 & Fig. S2). This provided additional constraints for model calibration.

There are multiple aspects of projected climate change (increases in salinity, temperature, and carbon dioxide) and human-induced modifications (decreases in suspended sediment over time and nutrient enrichment), that are not factored explicitly into MEM. Although salinity is not expected to increase drastically in the San Francisco Bay Estuary [17], increases on the order of five to seven can reduce biomass and diversity, especially in low salinity brackish and freshwater sites [49], [52]. Further complexity is added with changes in freshwater flow that are strongly influenced by snow runoff magnitude and season [69], which are not necessarily a result of climate change. Incorporating a function into MEM that reduces peak biomass over time could be a way of addressing shifting salinity dynamics without affecting the complexity of the model. The MEM does not model century-level changes in temperature and the resulting increase [20], [42] or decrease due to aridity [70] in productivity that has been documented with some marsh plant species, and could depend largely on marsh type and climate. However, to date, few field studies have occurred across a broad array of marsh species to address the magnitude of change. Human induced modifications to sediment supply and nutrient enrichment could also affect marsh resiliency. Implementing a decay curve on the concentration of suspended sediments could allow the model to begin with current values that decay over time in accordance with the uncertainty in concentrations in the future [17]. Effects of nutrient enrichment on marsh stability are mixed [71], and therefore would be difficult to incorporate into MEM.

The MEM is a zero-dimensional model that forecasts changes in elevation at a single point. Although the results can be applied to a digital elevation model as was done in this study, MEM is not inherently a spatially-explicit model. Landscape context (i.e., channel proximity, neighbor influence) is not taken into consideration when point-based results are applied spatially, nor are the effects of wind/wave erosion, which have been shown to strongly influence marsh stability [72]–[74]. A more realistic model of sediment dynamics would incorporate declining sediment concentration (conservation of mass), and hence differential deposition with distance from the source channel [63] and erosion near channel and bay edges. Model predictions should be most reliable in the local vicinity of calibration sites due to the assumption that sediment concentration is uniform across the marsh. Moreover, in the absence of a sediment mass balance, a calibration site in the marsh interior is preferable to one close to a creek bank.

### Conclusions

Across a range of century SLR rates, we demonstrated the important role of plant productivity on marsh resiliency. The tidal wetlands remained resilient to the pressures of increased sea level until reaching a tipping point where accommodation space, specifically adjacent upland habitat, was needed for maintenance of marsh habitat [6]. In all cases when the SLR rate was 100 cm/century or more, the majority of the marsh plain was at elevations characteristic of low marsh plant communities or lower. With the diverse array of resident bird and mammal species that utilize the mid and high marsh [75], particularly nesting birds, the loss of high elevation refugia could lead to a reduction in wildlife populations [76]. The sites that have adjacent upland areas were able to gain new mid/high marsh habitat at the highest rate of SLR, which increases the area of high elevation refugia. Up to a certain point, marshes can maintain vegetated elevations with increasing SLR, but accretion alone is not enough to support marsh habitat under the bleakest of scenarios. Site managers and agencies should identify and secure key upland locations near current marshes in order to allow marsh migration to occur.

## Supporting Information

Figure S1 **Coon Island plant biomass histogram.** Histogram of plant biomass occurrences at Coon Island that was used to determine peak biomass.
(TIF)

Figure S2 **Modeled versus measured soil bulk density and percent organic matter.** Comparison of modeled soil bulk density and percent organic matter with depth to soil core data (Callaway et al. 2012) collected at each site.
(TIF)

Figure S3 **Change in habitat cover under 24 cm/century at all marshes.** Change in percent cover of each habitat type over time for each suspended sediment concentration with 24 cm/century sea-level rise for all sites, with elevations color-coded to indicate unvegetated (brown), low marsh (light green), mid/high marsh (medium green), and upland (beige) areas.
(TIF)

Figure S4 **Habitat distributions at Rush Ranch at mid suspended sediment concentrations.** Distribution of modeled marsh habitat types in 2110 at Rush Ranch with 52 cm/century, 100 cm/century, and 180 cm/century sea-level rise at mid suspended sediment concentrations.
(TIF)

Table S1 **Marsh Equilibrium Model inputs for each tidal marsh.**
(DOCX)

Table S2 **Comparison of accretion rate and mineral accumulation between marsh soil cores** [**39**] **and MEM model results at comparable elevations at each site.**
(DOCX)
