# Supplementary figures and images for: Modeling Tidal Marsh Distribution with Sea-Level Rise: Evaluating the Role of Vegetation, Sediment, and Upland Habitat in Marsh Resiliency
Source: PLoS One. 2014 Feb 13;9(2):e88760. doi: 10.1371/journal.pone.0088760 (PMC3923833; doi:10.1371/journal.pone.0088760)

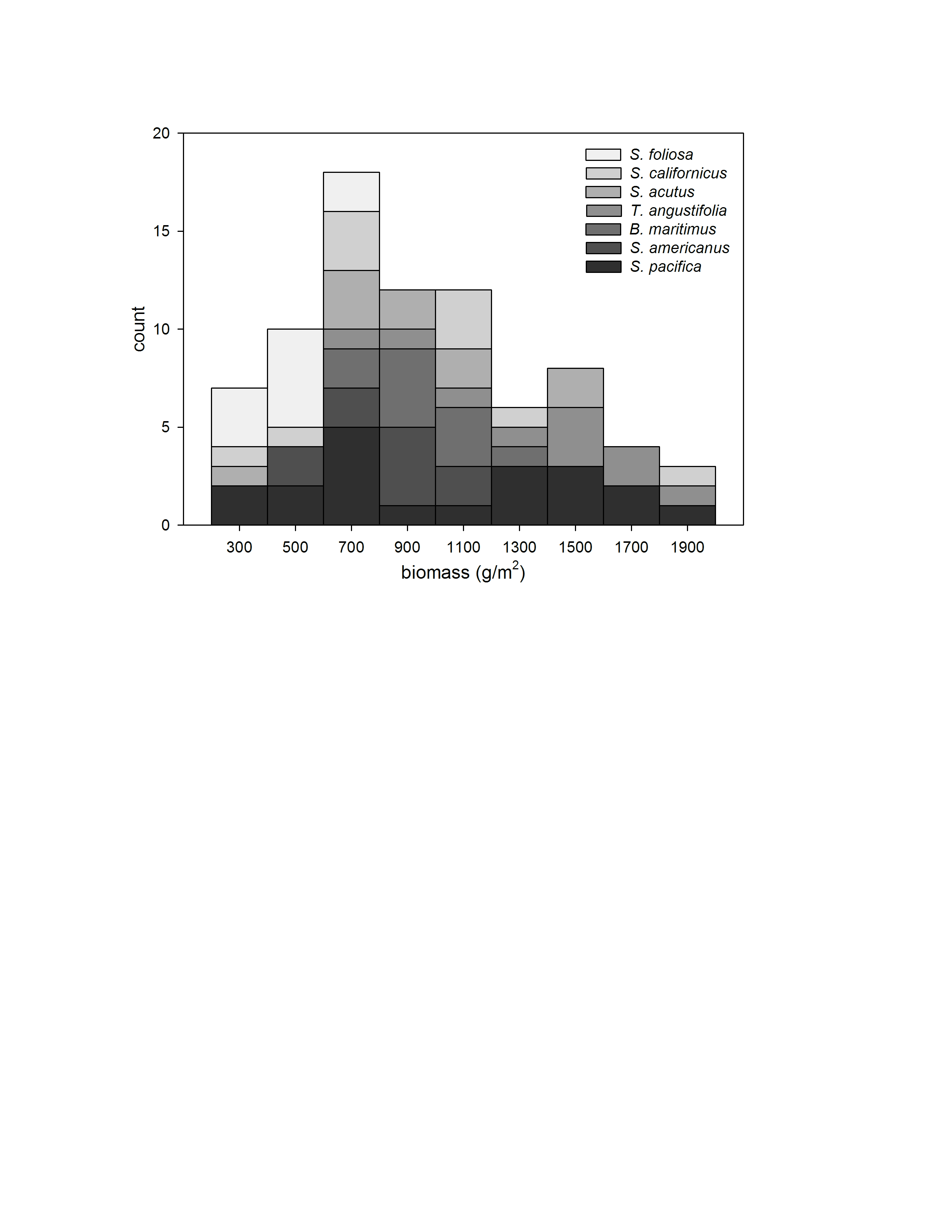

Supplement: Figure S1 — Coon Island plant biomass histogram. Histogram of plant biomass occurrences at Coon Island that was used to determine peak biomass. (TIF) [file pone.0088760.s001.tif]

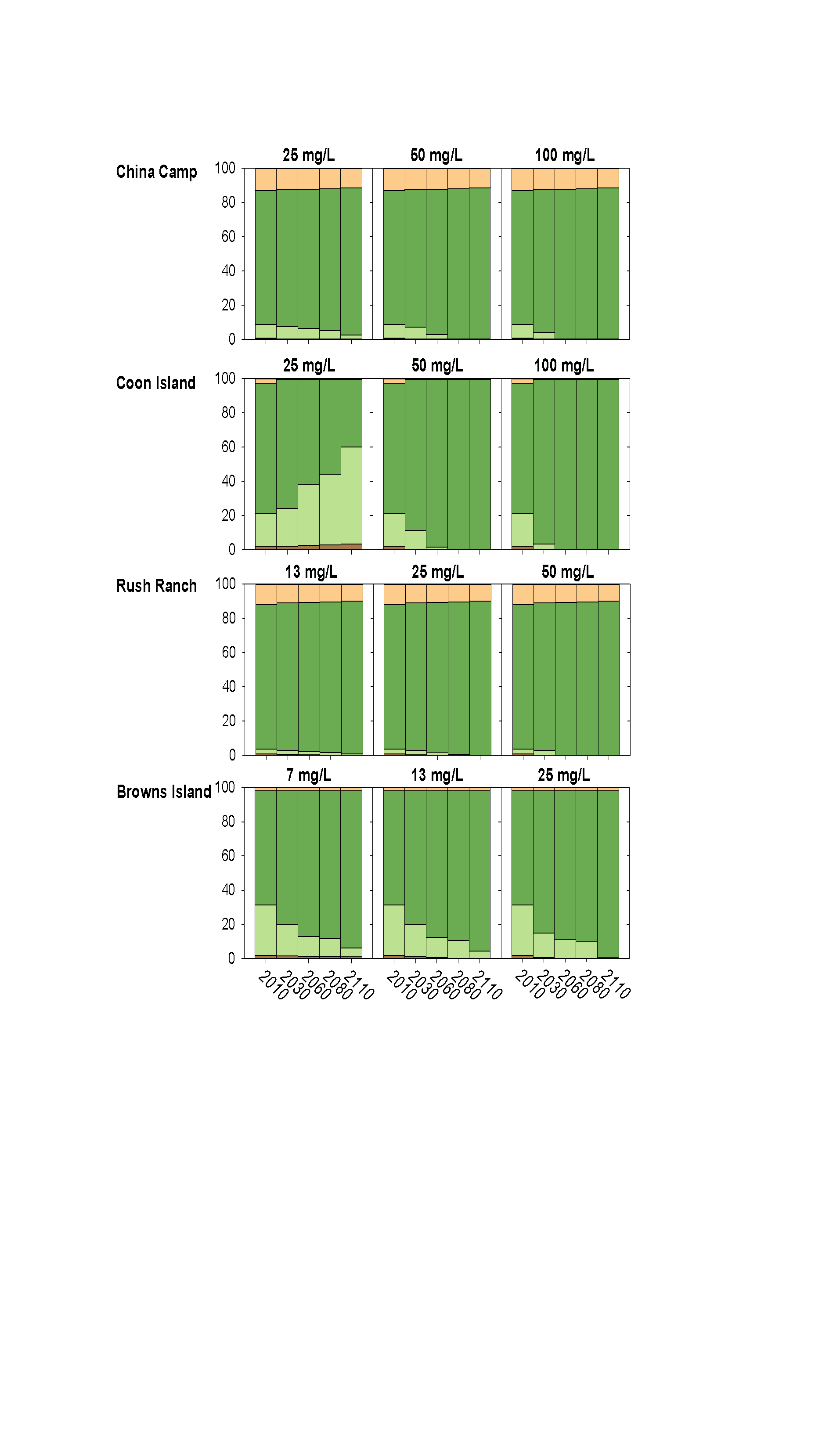

Supplement: Figure S3 — Change in habitat cover under 24 cm/century at all marshes. Change in percent cover of each habitat type over time for each suspended sediment concentration with 24 cm/century sea-level rise for all sites, with elevations color-coded to indicate unvegetated (brown), low marsh (light green), mid/high marsh (medium green), and upland (beige) areas. (TIF) [file pone.0088760.s003.tif]

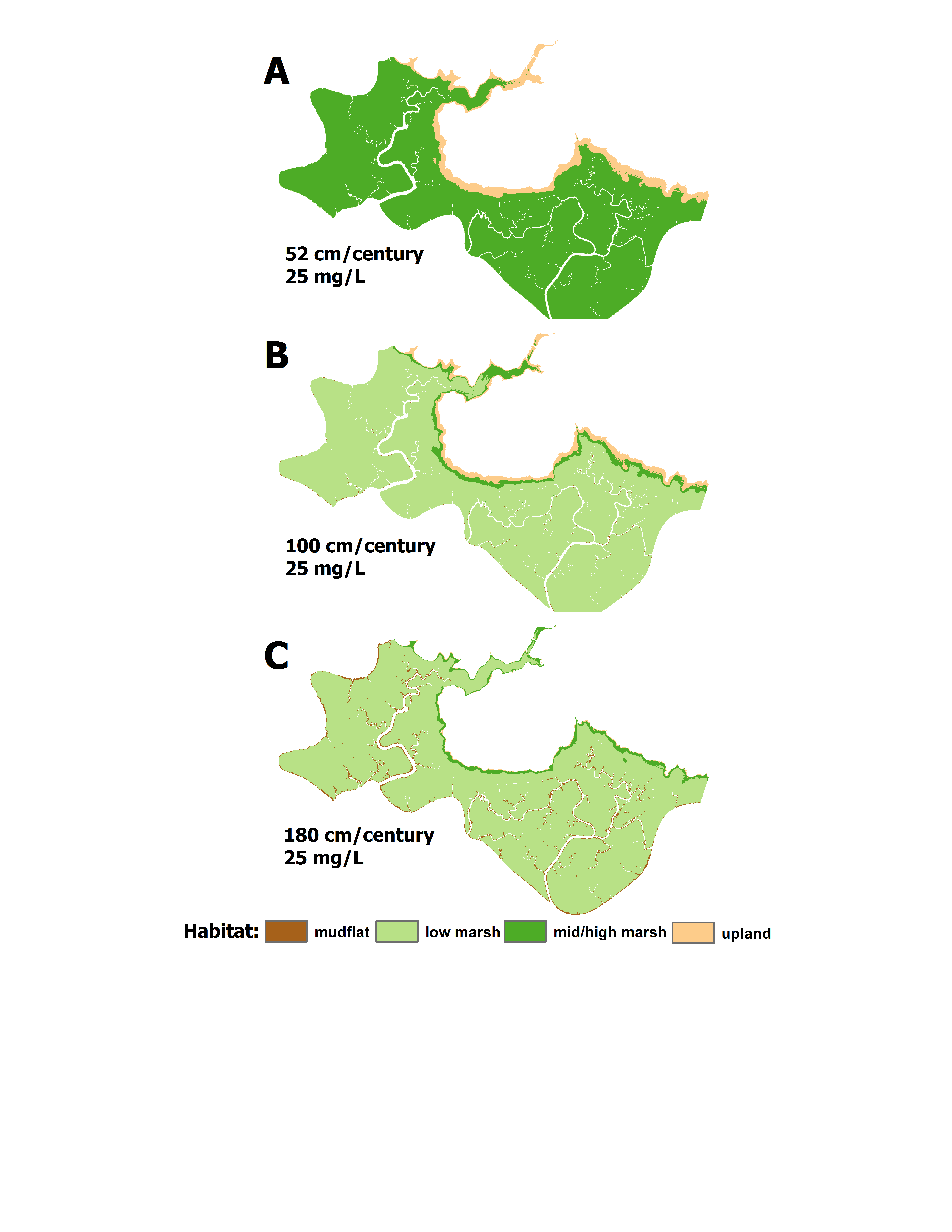

Supplement: Figure S4 — Habitat distributions at Rush Ranch at mid suspended sediment concentrations. Distribution of modeled marsh habitat types in 2110 at Rush Ranch with 52 cm/century, 100 cm/century, and 180 cm/century sea-level rise at mid suspended sediment concentrations. (TIF) [file pone.0088760.s004.tif]
