# Supplementary material for: Modeling Tidal Marsh Distribution with Sea-Level Rise: Evaluating the Role of Vegetation, Sediment, and Upland Habitat in Marsh Resiliency
Source: PLoS One. 2014 Feb 13;9(2):e88760. doi: 10.1371/journal.pone.0088760 (PMC3923833; doi:10.1371/journal.pone.0088760)
Supplement: Table S1 — Marsh Equilibrium Model inputs for each tidal marsh. (DOCX) [file pone.0088760.s005.docx]

Table S1. Marsh Equilibrium Model inputs for each tidal marsh.

|  | Site | | | |
| --- | --- | --- | --- | --- |
|  | China Camp | Coon Island | Rush Ranch | Browns Island |
| *mean higher high water (cm NAVD88)* | 191 | 198 | 198 | 190 |
| *mean sea level (cm NAVD88)* | 106 | 110 | 110 | 110 |
| *suspended sediment concentration (mg/l)* | 100, 50, 25 | 100, 50, 25 | 50, 25, 13 | 25, 13, 7 |
| *max. vegetation elevation (cm NAVD88)* | 195 | 200 | 200 | 195 |
| *min. vegetation elevation (cm NAVD88)* | 70 | 80 | 80 | 80 |
| *elevation of peak biomass (cm NAVD88)* | 170 | 160 | 170 | 160 |
| *max. biomass (g/m^2^)* | 1200 | 1700 | 2400 | 2500 |
| *organic matter decay rate (yr^-1^)* | -0.3 | -0.25 | -0.2 | -0.2 |
| *root to shoot ratio (g/g)* | 2.5 | 2.9 | 3 | 2.5 |
| *refractory carbon fraction, kr (g/g)* | 0.1 | 0.1 | 0.09 | 0.1 |
| *below-ground turnover rate (yr^-1^)* | 1 | 1 | 1 | 1 |
| *max (95%) root depth (cm)* | 20 | 25 | 40 | 40 |
