# Supplementary material for: Modeling Tidal Marsh Distribution with Sea-Level Rise: Evaluating the Role of Vegetation, Sediment, and Upland Habitat in Marsh Resiliency
Source: PLoS One. 2014 Feb 13;9(2):e88760. doi: 10.1371/journal.pone.0088760 (PMC3923833; doi:10.1371/journal.pone.0088760)
Supplement: Table S2 — Comparison of accretion rate and mineral accumulation between marsh soil cores [39] and MEM model results at comparable elevations at each site. (DOCX) [file pone.0088760.s006.docx]

Table S2. Comparison of accretion rate and mineral accumulation between marsh soil cores [[39](#_ENREF_39)] and MEM model results at comparable elevations at each site.

|  | accretion rate (cm yr^-1^) | | |  | mineral accumulation (g m^-2^ yr^-1^) | | |
| --- | --- | --- | --- | --- | --- | --- | --- |
|  | soil core values |  | model results |  | soil core values |  | model results |
| China Camp | 0.31-0.43 |  | 0.32 |  | 966-1972 |  | 1419 |
| Coon Island | 0.11-0.32 |  | 0.23 |  | 301-839 |  | 801 |
| Rush Ranch | 0.2-0.29 |  | 0.25 |  | 312-623 |  | 338 |
| Browns Island | 0.2-0.27 |  | 0.26 |  | 189-513 |  | 260 |
